# Supplementary figures and images for: Transcriptional control of motor pool formation and motor circuit connectivity by the LIM-HD protein Isl2
Source: eLife. 2023 Oct 23;12:e84596. doi: 10.7554/eLife.84596 (PMC10637776; doi:10.7554/eLife.84596)

Figure 3-figure supplement 1

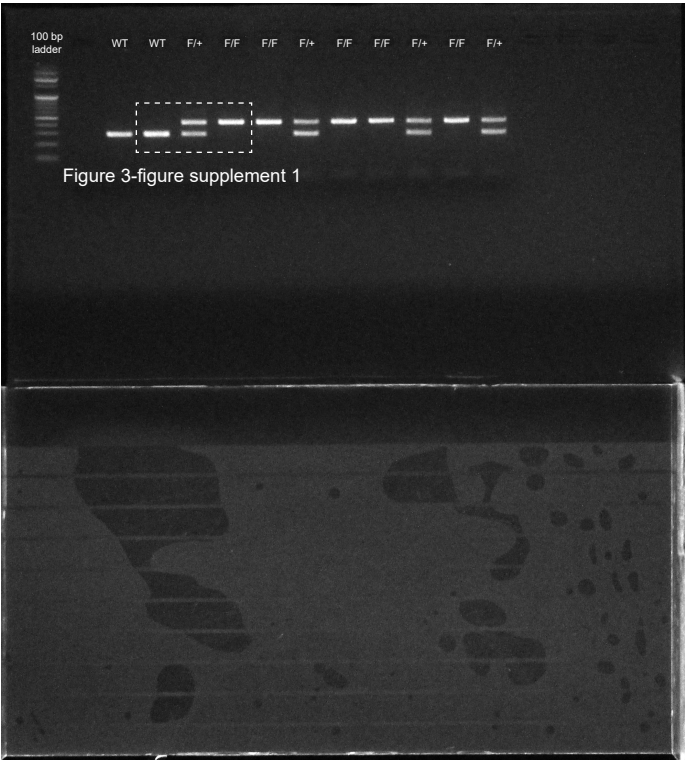

Supplement: Figure 3—figure supplement 1—source data 1. [file elife-84596-fig3-figsupp1-data1.zip › Figure 3-figure supplement 1-source data 1/Figure 3-figure supplement 1-source data 1.pdf]

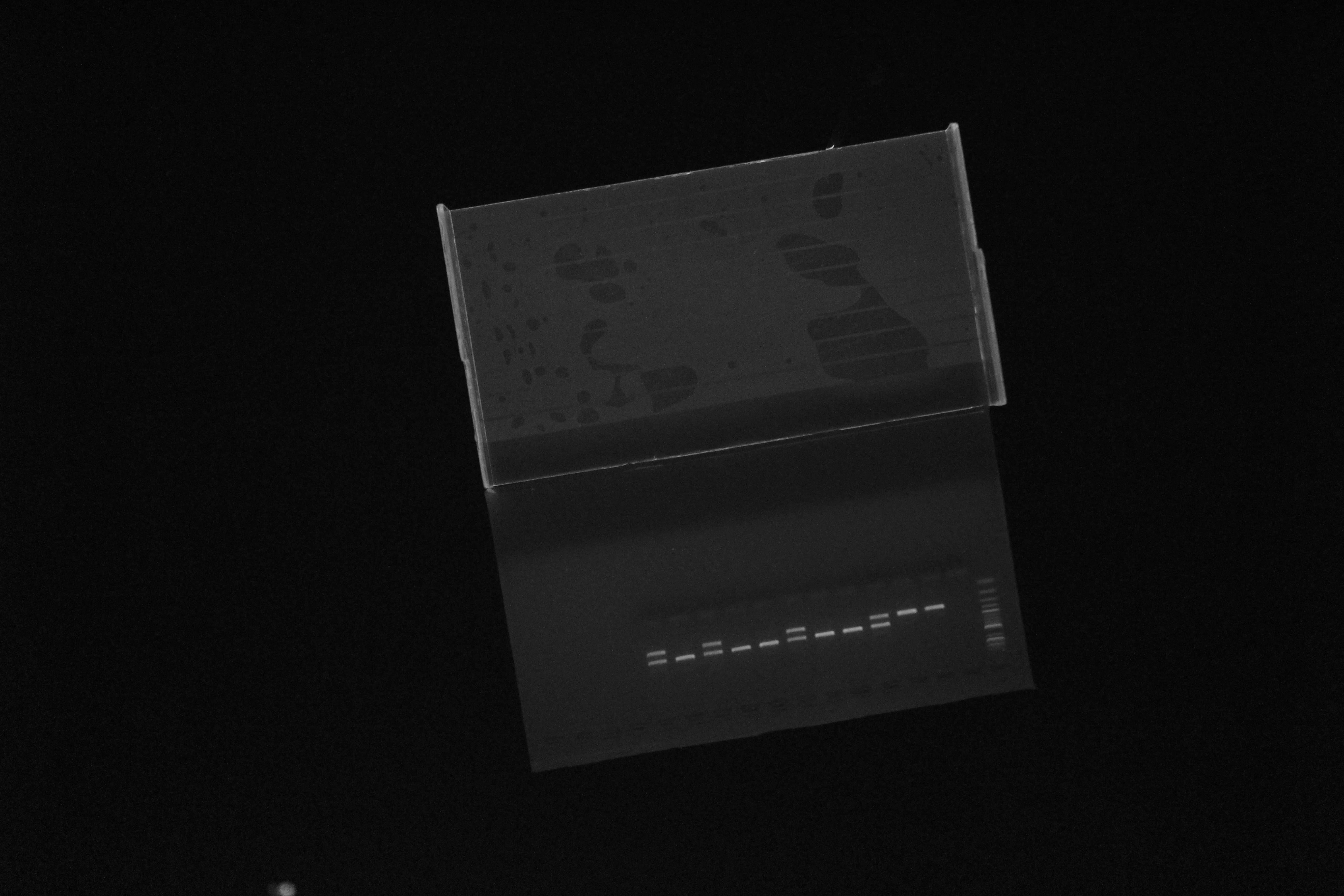

Supplement: Figure 3—figure supplement 1—source data 1. [file elife-84596-fig3-figsupp1-data1.zip › Figure 3-figure supplement 1-source data 1/Figure 3-figure supplement 1-source data 1_unedited.JPG]

Figure 5b

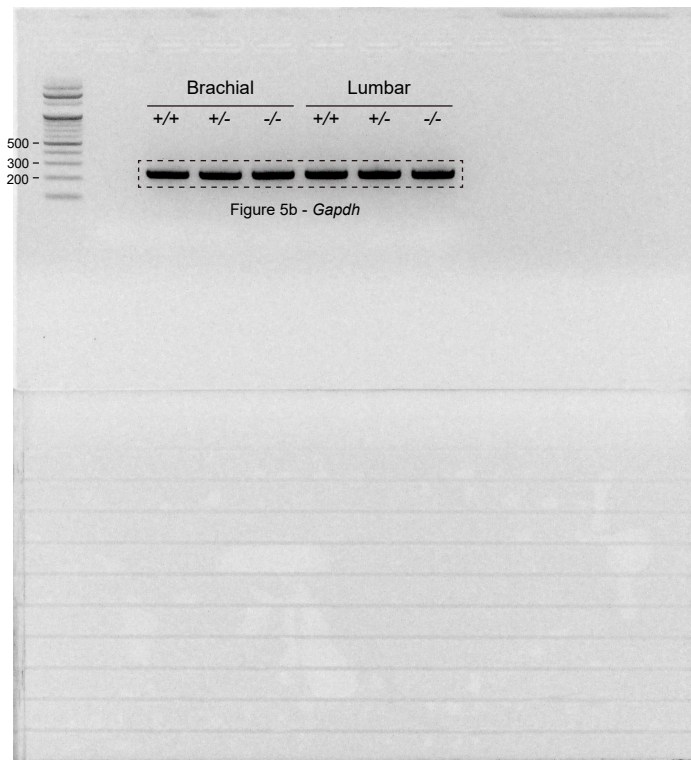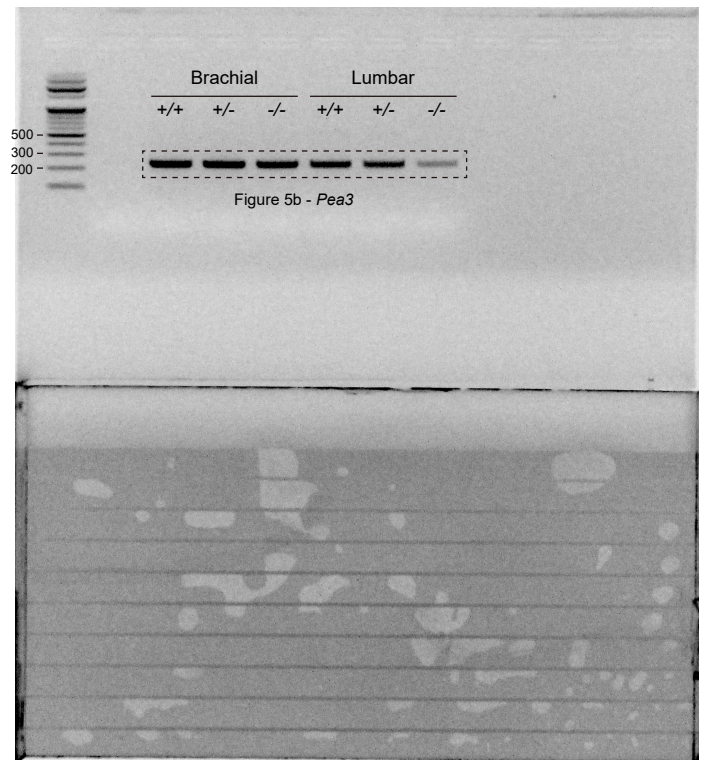

Supplement: Figure 5—source data 3. [file elife-84596-fig5-data3.zip › Figure 5–source data 3/Figure 5-source data 3.pdf]

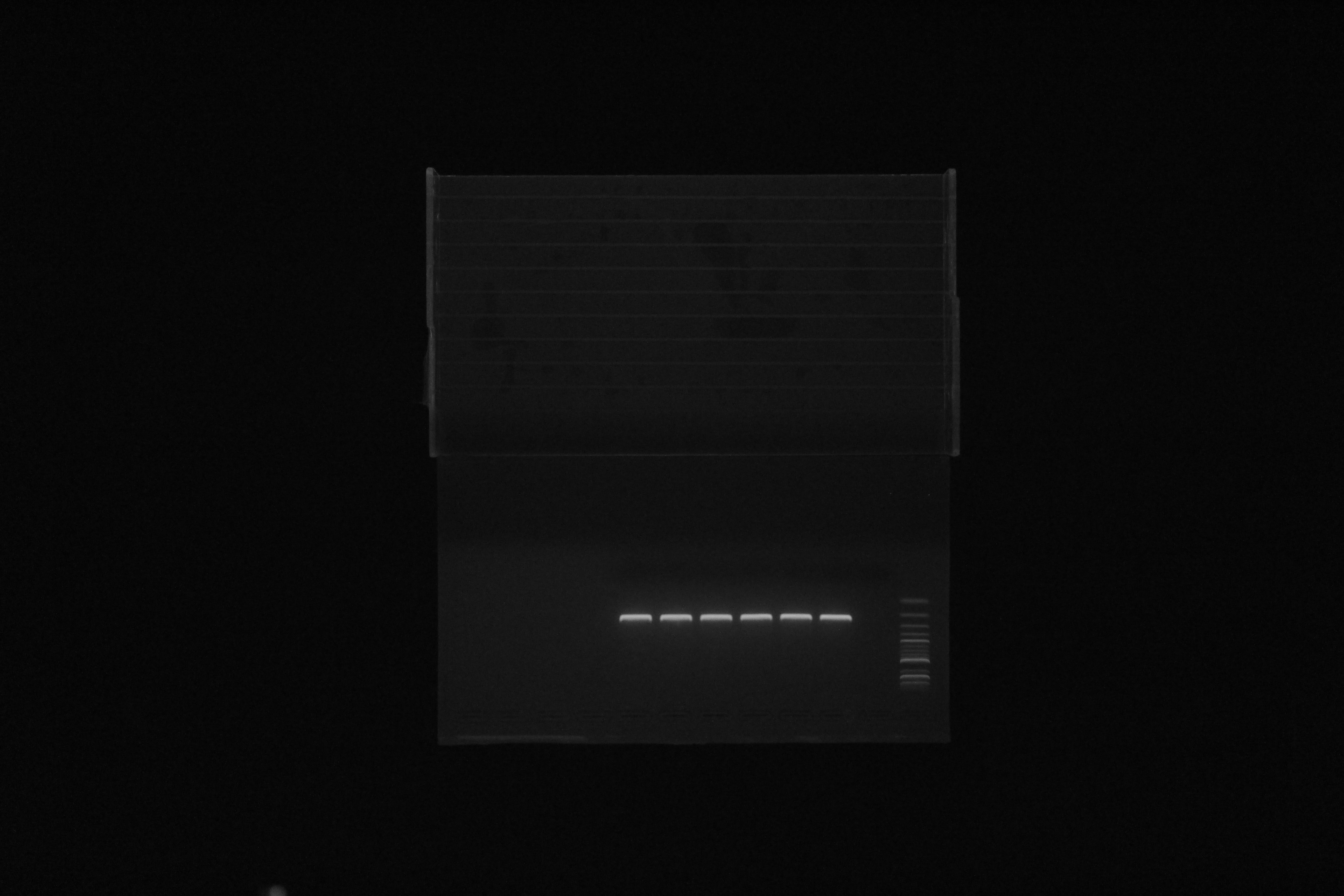

Supplement: Figure 5—source data 3. [file elife-84596-fig5-data3.zip › Figure 5–source data 3/Figure 5-source data 3_unedited-Gapdh.JPG]

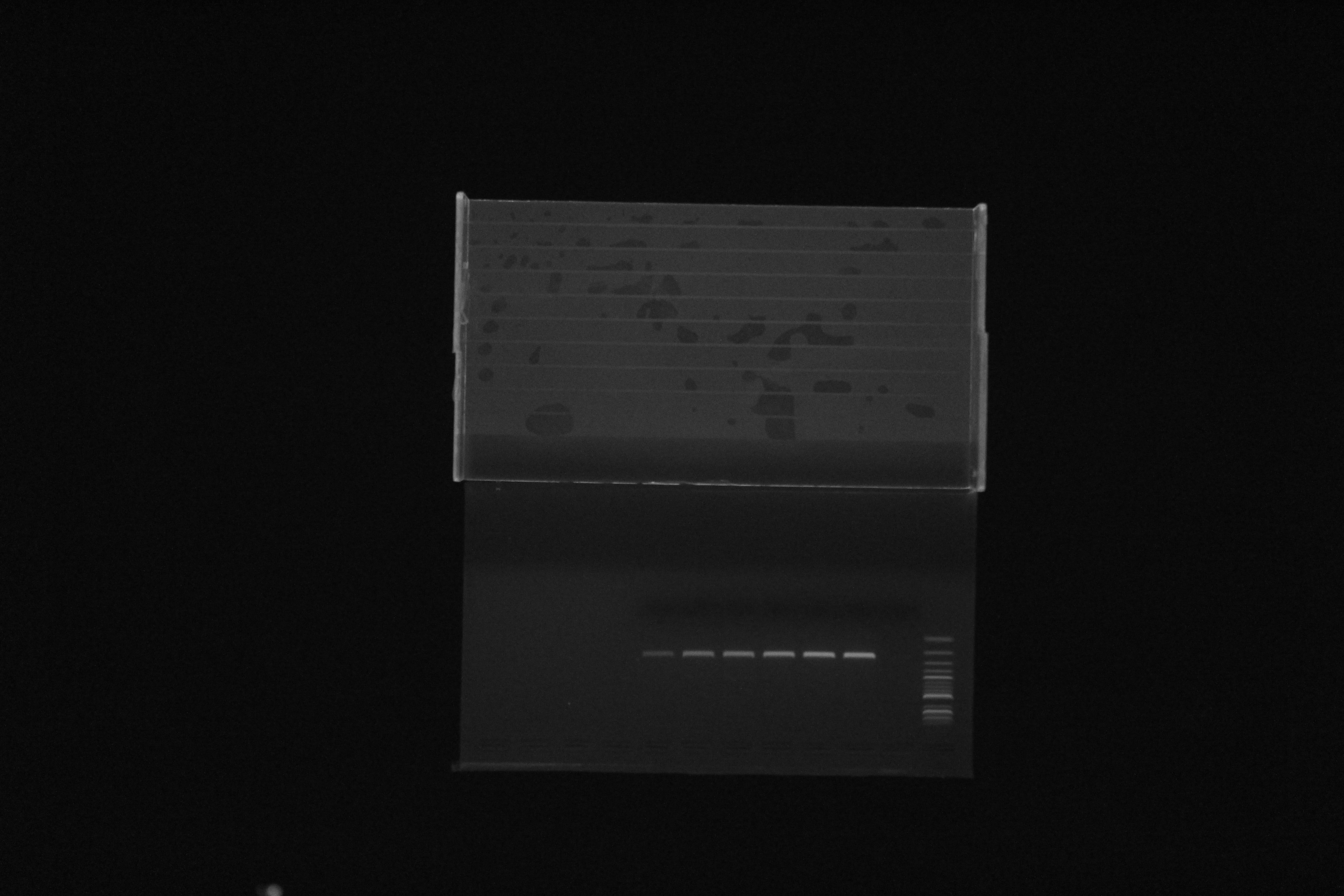

Supplement: Figure 5—source data 3. [file elife-84596-fig5-data3.zip › Figure 5–source data 3/Figure 5-source data 3_unedited-Pea3.JPG]

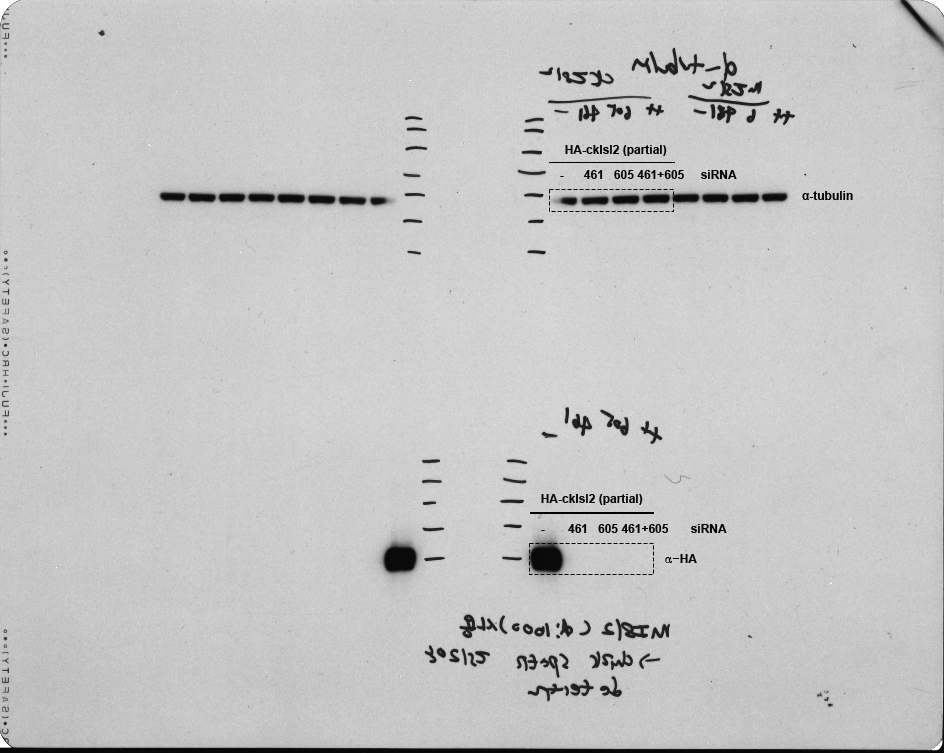

Supplement: Figure 5—figure supplement 2—source data 1. [file elife-84596-fig5-figsupp2-data1.zip › Figure 5-figure supplement 2-source data 1/Figure 5-figure supplement 2-source data 1_labeled.tif]

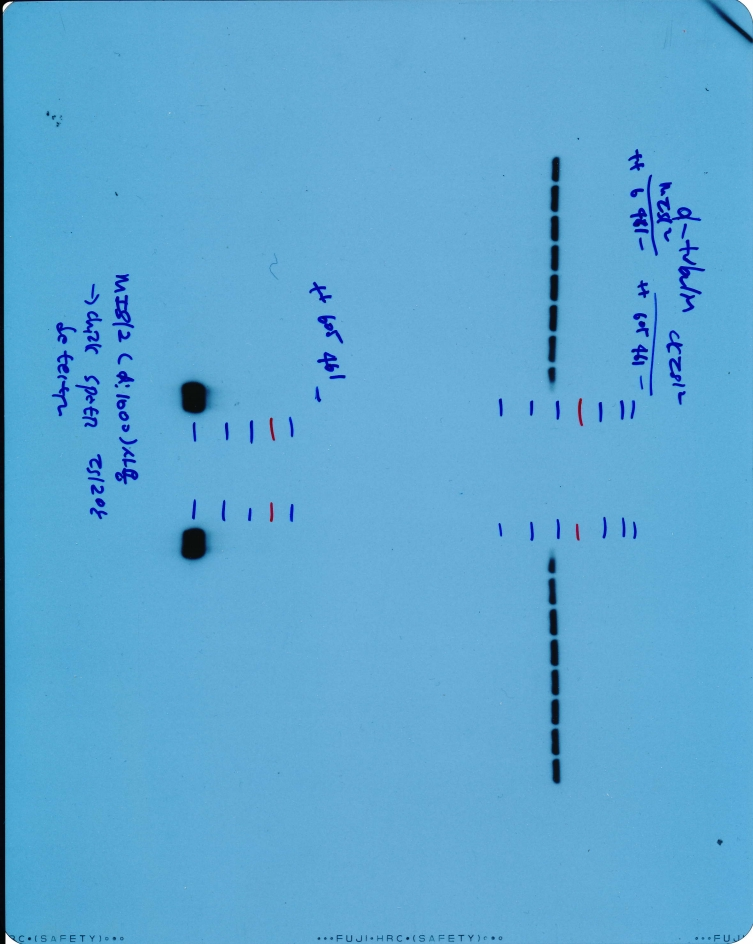

Supplement: Figure 5—figure supplement 2—source data 1. [file elife-84596-fig5-figsupp2-data1.zip › Figure 5-figure supplement 2-source data 1/Figure 5-figure supplement 2-source data 1_raw unedited.tiff]
