# Supplementary material for: Transcriptional control of motor pool formation and motor circuit connectivity by the LIM-HD protein Isl2
Source: eLife. 2023 Oct 23;12:e84596. doi: 10.7554/eLife.84596 (PMC10637776; doi:10.7554/eLife.84596)
Supplement: Supplementary file 1. — Genotype distribution of Isl2-null and conditional KO mice. Expected Mendelian inheritance and observed inheritance after breeding between Isl2 heterozygotes or between Isl2 +/-; Olig2Cre and Isl2F/F. p-Values of the Mendelian ratios were calculated using chi-square tests. [file elife-84596-supp1.docx]

**Supplementary File 1. Genotype distribution of *Isl2* KO and conditional KO mice**

| Offspring | | | | | |
| --- | --- | --- | --- | --- | --- |
| Age | Genotype | No. Expected | No. Observed | Chi-square value | *P* |
| Embryonic stages | *Isl2^+/+^* | 77 (25%) | 86 (28%) | 7.739 | 0.0209 |
|  | *Isl2^+/−^* | 153 (50%) | 129 (42%) |  |  |
|  | *Isl2^−/−^* | 77 (25%) | 92 (30%) |  |  |
|  | Total | 307 | 307 |  |  |
| Postnatal stages | *Isl2^+/+^* | 108 (25%) | 131 (30%) | 43.102 | < 0.0001 |
|  | *Isl2^+/−^* | 217 (50%) | 253 (58%) |  |  |
|  | *Isl2^−/−^* | 108 (25%) | 49 (11%) |  |  |
|  | Total | 433 | 433 |  |  |
| Embryonic stages | *Isl2^F/+^* | 21 (25%) | 20 (25%) | 0.498 | 0.9194 |
|  | *Isl2^F/KO^* | 20 (25%) | 22 (27%) |  |  |
|  | *Isl2^F/+^*; Olig2-Cre | 20 (25%) | 18 (22%) |  |  |
|  | *Isl2^F/KO^*; Olig2-Cre | 20 (25%) | 21 (26%) |  |  |
|  | Total | 81 | 81 |  |  |
| Postnatal stages | *Isl2^F/+^* | 18 (25%) | 29 (42%) | 25.840 | < 0.0001 |
|  | *Isl2^F/KO^* | 17 (25%) | 23 (33%) |  |  |
|  | *Isl2^F/+^*; Olig2-Cre | 17 (25%) | 17 (25%) |  |  |
|  | *Isl2^F/KO^*; Olig2-Cre | 17 (25%) | 0 (0%) |  |  |
|  | Total | 69 | 69 |  |  |

Genotype distribution of *Isl2* null and conditional KO mice. Expected Mendelian inheritance and observed inheritance after breeding between *Isl2* heterozygotes or between *Isl2^+/−^*; Olig2-Cre and *Isl2^F/F^*. *P*-values of the Mendelian ratios were calculated using chi-square tests.
