# Supplementary material for: Transcriptional control of motor pool formation and motor circuit connectivity by the LIM-HD protein Isl2
Source: eLife. 2023 Oct 23;12:e84596. doi: 10.7554/eLife.84596 (PMC10637776; doi:10.7554/eLife.84596)
Supplement: Supplementary file 2. — Experimental sample sizes for figures and figure supplements. [file elife-84596-supp2.docx]

**Supplementary File 2. Experimental sample sizes**

| **Figure 1** | | |
| --- | --- | --- |
| (d) | E10.5 | n = 6 sections per level, 2 animals |

| **Figure 2** | | | |
| --- | --- | --- | --- |
| (c) | Brachial | Average from n = 3 mice, 4-6 sections each animal | |
|  | Lumbar | Average from n = 3 mice, 8-16 sections each animal | |
|  | Thoracic | Average from n = 3 mice, 16-24 sections each animal | |
| **Figure 2-figure supplement 1** | | | |
| (c) | E13.5, lumbar | | Average from n = 3 mice, 2 sections each animal |

| **Figure 3** | | |
| --- | --- | --- |
| (b) | C6 | Average from n = 3 mice, 6 sections per group |
|  | C8 | Average from n = 3 mice, 6 sections per group |
| (c) | CM | Average from n = 3 mice, 12 sections per group |
|  | LD | Average from n = 3 mice, 12 sections per group |
|  | FCU | Average from n = 3 mice, 6 sections per group |
| **Figure 3-figure supplement 2, 3** | | |
| (a) | C6, Het | n = 6 section per group, 3 animals  794 cells for Hb9^+^Lhx3^-^ MNs (somatic MNs); 397 cells for Hb9^+^Lhx3^+^ (MMC); 667 cells for Foxp1^+^ (LMC); 190 cells for Hb9::GFP^+^Foxp1^-^Scip^+^ (MMC/HMC); 909 cells for Hb9::GFP^+^Isl1^+^ (MMC and LMCm); 369 cells for Hb9::GFP^+^Lhx1^+^ (LMCl); 164 cells for Hb9::GFP^+^Isl1^+^Etv4^+^ (CM); 128 cells for Hb9::GFP^+^Isl1^-^Etv4^+^ (LD) |
|  | C6, KO | n = 6 section per group, 3 animals  661 cells for Hb9^+^Lhx3^-^ MNs (somatic MNs); 372 cells for Hb9^+^Lhx3^+^ (MMC); 610 cells for Foxp1^+^ (LMC); 100 cells for Hb9::GFP^+^Foxp1^-^Scip^+^ (MMC/HMC); 642 cells for Hb9::GFP^+^Isl1^+^ (MMC and LMCm); 391 cells for Hb9::GFP^+^Lhx1^+^ (LMCl); 180 cells for Hb9::GFP^+^Isl1^+^Etv4^+^ (CM); 124 cells for Hb9::GFP^+^Isl1^-^Etv4^+^ (LD) |
|  | C6, cKO | n = 6 section per group, 3 animals  612 cells for Hb9^+^Lhx3^-^ MNs (somatic MNs); 323 cells for Hb9^+^Lhx3^+^ (MMC); 559 cells for Foxp1^+^ (LMC); 105 cells for Isl1^+^Foxp1^-^Scip^+^ (MMC/HMC); 569 cells for Hb9^+^Isl1^+^ (MMC and LMCm); 292 cells for Hb9^+^Lhx1^+^ (LMCl); 151 cells for Hb9^+^Isl1^+^Etv4^+^ (CM); 104 cells for Hb9^+^Isl1^-^Etv4^+^ (LD) |
|  | C8, Het | n = 6 section per group, 3 animals  736 cells for Hb9^+^Lhx3^-^ MNs (somatic MNs); 319 cells for Hb9^+^Lhx3^+^ (MMC); 290 cells for Foxp1^+^ (LMC); 94 cells for Hb9::GFP^+^Foxp1^-^Scip^+^ (MMC/HMC); 219 cells for Foxp1^+^Scip^+^ (FCU); 940 cells for Hb9::GFP^+^Isl1^+^ (MMC and LMCm); 243 cells for Hb9::GFP^+^Lhx1^+^ (LMCl); 281 cells for Hb9::GFP^+^Isl1^+^Etv4^+^ (CM); 62 cells for Hb9::GFP^+^Isl1^-^Etv4^+^ (LD) |
|  | C8, KO | n = 6 section per group, 3 animals  702 cells for Hb9^+^Lhx3^-^ MNs (somatic MNs); 398 cells for Hb9^+^Lhx3^+^ (MMC); 326 cells for Foxp1^+^ (LMC); 63 cells for Hb9::GFP^+^Foxp1^-^Scip^+^ (MMC/HMC); 185 cells for Foxp1^+^Scip^+^ (FCU); 640 cells for Hb9::GFP^+^Isl1^+^ (MMC and LMCm); 274 cells for Hb9::GFP^+^Lhx1^+^ (LMCl); 200 cells for Hb9::GFP^+^Isl1^+^Etv4^+^ (CM); 72 cells for Hb9::GFP^+^Isl1^-^Etv4^+^ (LD) |
|  | C8, cKO | n = 6 section per group, 3 animals  614 cells for Hb9^+^Lhx3^-^ MNs (somatic MNs); 342 cells for Hb9^+^Lhx3^+^ (MMC); 311 cells for Foxp1^+^ (LMC); 76 cells for Isl1^+^Foxp1^-^Scip^+^ (MMC/HMC); 182 cells for Foxp1^+^Scip^+^ (FCU); 569 cells for Hb9^+^Isl1^+^ (MMC and LMCm); 194 cells for Hb9^+^Lhx1^+^ (LMCl); 180 cells for Hb9^+^Isl1^+^Etv4^+^ (CM); 71 cells for Hb9^+^Isl1^-^Etv4^+^ (LD) |

| **Figure 4** | | |  |
| --- | --- | --- | --- |
| (b) | L2 | Average from n = 3 mice, 6 sections per group |  |
|  | L4 | Average from n = 3 mice, 6 sections per group |  |
| (c) | Etv4^+^ | Average from n = 3 mice, 20-48 sections per group |  |
|  | Nkx6.1^+^Lhx1^+^ | Average from n = 3 mice, 8-12 sections per group |  |
| **Figure 4-figure supplement 1, 2** | | | |
| (a) | L2, Het | n = 6 section per group, 3 animals  549 cells for Hb9^+^Lhx3^-^ MNs (somatic MNs); 221 cells for Hb9^+^Lhx3^+^ (MMC); 609 cells for Foxp1^+^ (LMC); 191 cells for Hb9::GFP^+^Foxp1^-^Scip^+^ (MMC/HMC); 398 cells for Hb9::GFP^+^Nkx6.1^+^ (MMC and LMCm); 214 cells for Hb9::GFP^+^Lhx1^+^ (LMCl); 128 cells for Hb9::GFP^+^Etv4^+^ (Rf/Tfl, Gl) | |
|  | L2, KO | n = 6 section per group, 3 animals  710 cells for Hb9^+^Lhx3^-^ MNs (somatic MNs); 265 cells for Hb9^+^Lhx3^+^ (MMC); 630 cells for Foxp1^+^ (LMC); 186 cells for Hb9::GFP^+^Foxp1^-^Scip^+^ (MMC/HMC); 395 cells for Hb9::GFP^+^Nkx6.1^+^ (MMC and LMCm); 328 cells for Hb9::GFP^+^Lhx1^+^ (LMCl); 33 cells for Hb9::GFP^+^Etv4^+^ (Rf/Tfl, Gl) | |
|  | L2, cKO | n = 6 section per group, 3 animals  621 cells for Hb9^+^Lhx3^-^ MNs (somatic MNs); 255 cells for Hb9^+^Lhx3^+^ (MMC); 766 cells for Foxp1^+^ (LMC); 237 cells for Isl1^+^Foxp1^-^Scip^+^ (MMC/HMC); 377 cells for Hb9^+^Nkx6.1^+^ (MMC and LMCm); 302 cells for Hb9^+^Lhx1^+^ (LMCl); 20 cells for Hb9^+^Etv4^+^ (Rf/Tfl, Gl) | |
|  | L4, Het | n = 6 section per group, 3 animals  579 cells for Hb9^+^Lhx3^-^ MNs (somatic MNs); 208 cells for Hb9^+^Lhx3^+^ (MMC); 683 cells for Foxp1^+^ (LMC); 180 cells for Hb9::GFP^+^Foxp1^-^Scip^+^ (MMC/HMC); 576 cells for Hb9::GFP^+^Nkx6.1^+^ (MMC and LMCm); 312 cells for Hb9::GFP^+^Lhx1^+^ (LMCl); 58 cells for Hb9::GFP^+^Nkx6.1^+^Lhx1^+^ (Ta); 89 cells for Hb9::GFP^+^Etv4^+^ (Rf/Tfl, Gl) | |
|  | L4, KO | n = 6 section per group, 3 animals  698 cells for Hb9^+^Lhx3^-^ MNs (somatic MNs); 215 cells for Hb9^+^Lhx3^+^ (MMC); 718 cells for Foxp1^+^ (LMC); 201 cells for Hb9::GFP^+^Foxp1^-^Scip^+^ (MMC/HMC); 476 cells for Hb9::GFP^+^Nkx6.1^+^ (MMC and LMCm); 466 cells for Hb9::GFP^+^Lhx1^+^ (LMCl); 91 cells for Hb9::GFP^+^Nkx6.1^+^Lhx1^+^ (Ta); 16 cells for Hb9::GFP^+^Etv4^+^ (Rf/Tfl, Gl) | |
|  | L4, cKO | n = 6 section per group, 3 animals  650 cells for Hb9^+^Lhx3^-^ MNs (somatic MNs); 223 cells for Hb9^+^Lhx3^+^ (MMC); 865 cells for Foxp1^+^ (LMC); 172 cells for Isl1^+^Foxp1^-^Scip^+^ (MMC/HMC); 649 cells for Hb9^+^Nkx6.1^+^ (MMC and LMCm); 514 cells for Hb9^+^Lhx1^+^ (LMCl); 136 cells for Hb9^+^Nkx6.1^+^Lhx1^+^ (Ta); 12 cells for Hb9^+^Etv4^+^ (Rf/Tfl, Gl) | |

| **Figure 5** | | |
| --- | --- | --- |
| (b) | *Etv4, Gapdh* | Brachial, n = 3 animals per group  Lumbar, n = 6-8 animals per group |
| (c) | MNR2 | Average from n = 4 embryos, 18 sections per group |
|  | Isl2 | Average from n = 4 embryos, 26 sections per group |
|  | *ETV4* | Average from n = 3 embryos, 7 sections per group |
|  | *ALDH1A2* | Average from n = 3 embryos, 6 sections per group |
| **Figure 5-figure supplement 1** | | |
| (a) | *Etv4, Gapdh* | Brachial, n = 3 animals per group  Lumbar, n = 6-8 animals per group |
| **Figure 5-figure supplement 2** | | |
| (a) | Western blot | n = 1 |

| **Figure 6** | | |
| --- | --- | --- |
|  | Bulk RNA-seq | n = 3 animals per group |

| **Figure 7** | | |
| --- | --- | --- |
| (b) | Gl | Average from n = 3 mice per group, 10-17 sections per animal |
| (c) | Gl | n = 18 section per group, 3 animals per group  Het, n = 477 ChAT^+^ MNs  KO, n = 557 ChAT^+^ MNs |
| (d) | Gl | n = 6 section per group, 3 animals |
|  | Tfl | n = 6 section per group, 3 animals |
|  | Rf | n = 8 section per group, 3 animals |
| (f), (g) | Gl | n = 10-21 section per group, 3 animals per group  Het, n = 43 Rh-Dex^+^ Gl motor pools  KO, n = 62 Rh-Dex^+^ Gl motor pools |
| (h) | Gl | n = 10 random fields per group, 3 animals |
| (j), (k) | Gl | Het, n = 19 Rh-Dex^+^ Gl motor pools, 3 animals  KO, n = 14 Rh-Dex^+^ Gl motor pools, 3 animals |

| **Figure 8** | | |
| --- | --- | --- |
| (b) | P0, Gl | n = 4 muscles, 3 animals per group |
| (c) | P0, Gl | n = 90-120 axons, n = 4 muscles, 3 animals per group |
| (d) | P0, Gl | n = 4-5 muscles, 3 animals per group |
| (e) | P0, Gl | n = 4-5 muscles, 3 animals per group |
| (f) | P14, Gl | n = 4 muscles, 3 animals per group |
| (g) | P14, Gl | Het, n = 46 100-μm endplates, 3 muscles, 3 animals  KO, n = 39 100-μm endplates, 3 muscles, 3 animals |
| (i) | P14, Tfl | n = 4 muscles, 3 animals per group |
| (j) | P28, Tfl | Het, n = 11 NMJs, 3 animals  KO, n = 13 NMJs, 3 animals |
| (l) | Gl | Het, n = 17 random fields, 3 animals  KO, n = 16 random fields, 3 animals |
| (m) | Gl | Het, n = 17 random fields, 3 animals  KO, n = 8 random fields, 3 animals |
| (n) | Gl | Het, n = 22 NMJs, 3 animals  KO, n = 11 NMJs, 3 animals |
| (o) | Gl | Het, n = 22 NMJs, 3 animals  KO, n = 11 NMJs, 3 animals |
| (p) | Gl | Het, n = 17 random fields, 3 animals  KO, n = 16 random fields, 3 animals |
| (q) | Gl | Het, n = 39 NMJs, 3 animals  KO, n = 15 NMJs, 3 animals |
| (r) | Gl | Het, n = 39 NMJs, 3 animals  KO, n = 15 NMJs, 3 animals |
| **Figure 8-figure supplement 1** | | |
| (a-c) | E11.5-E13.5 | n > 1–2 animals per group |
| (d) | E13.5 | n = 2 mice per group |
| **Figure 8-figure supplement 2** | | |
| (a-b) | cKO, E18.5 | n = 3 mice per group |
| **Figure 8-figure supplement 3** | | |
| (a) | P0 | n > 1–2 animals per group |
| (b) | P14 | n > 1–2 animals per group |

| **Figure 9** | | |
| --- | --- | --- |
| (c) | 3 months | Het = 5, KO = 8 animals |
| (e) | Whole body | Het = 5, KO = 4 animals |
|  | Gluteus | Het, n = 6 muscles from 5 animals  KO, n = 5 muscles from 4 animals |
|  | Rectus femoris | Het, n = 6 muscles from 5 animals  KO, n = 5 muscles from 4 animals |
| (f–j) | EMG | 2 parts of EMG signal from each animal,  Het = 3, KO = 3 animals |
| **Figure 9-figure supplement 1** | | |
| (a) | Skeletal analysis | WT = 3, KO = 1 animal |
| (b) | Tibia length | WT = 3, KO = 3 animals |

Experimental sample sizes for figures and figure supplements.
