# Supplementary material for: Transcriptional control of motor pool formation and motor circuit connectivity by the LIM-HD protein Isl2
Source: eLife. 2023 Oct 23;12:e84596. doi: 10.7554/eLife.84596 (PMC10637776; doi:10.7554/eLife.84596)
Supplement: Supplementary file 3. — (a) List of DEGs downregulated in Isl2-null brachial spinal cords. (b) List of DEGs downregulated in Isl2-null lumbar spinal cords. (c) List of top 30 enriched genes in sb.LMCl.2 subcluster. [file elife-84596-supp3.docx]

**Supplementary File 3. List of DEGs downregulated in *Isl2*-null brachial and lumbar spinal cords**

- Supplementary File 3a. List of DEGs downregulated in *Isl2* null brachial spinal cords.

| Gene symbol | Gene ID | log2.fc | log2.baseMean | raw.pval |
| --- | --- | --- | --- | --- |
| Tmem254c | 100039192 | -0.644098525 | 8.675006274 | 0.000539647 |
| Tmem254a | 66039 | -0.661055299 | 8.685585713 | 0.000491788 |
| 2310030G06Rik | 66952 | -1.400086517 | 4.997282947 | 0.003256271 |
| Rpl29 | 19944 | -0.636864613 | 12.83295571 | 0.001276703 |
| Duxbl3 | 100039293 | -0.573810365 | 7.969932007 | 0.025383162 |
| Gzmm | 16904 | -0.589508437 | 6.26165985 | 0.044270267 |
| 1700019D03Rik | 67080 | -0.701680159 | 5.9089368 | 0.03544027 |
| Mstn | 17700 | -1.621715632 | 4.411581999 | 0.006726793 |
| Uts2b | 224065 | -1.099655488 | 8.775225111 | 2.90068E-15 |
| Pts | 19286 | -0.620227864 | 10.28226173 | 3.43782E-09 |
| Duxbl1 | 278672 | -0.525658611 | 8.116214026 | 0.014342951 |
| Layn | 244864 | -1.045134732 | 6.617736282 | 0.000179776 |
| Ntf3 | 18205 | -0.525018613 | 9.42281858 | 7.10838E-06 |
| Hint3 | 66847 | -0.617393551 | 10.30431154 | 4.07737E-07 |
| Xrcc3 | 74335 | -1.174440087 | 5.2873576 | 0.010443029 |
| Kcnj12 | 16515 | -0.558525649 | 7.568385013 | 0.005331021 |
| Klf9 | 16601 | -0.703834144 | 6.907369341 | 0.021288184 |
| Nupr1l | 69034 | -1.191457108 | 5.030219562 | 0.017302656 |
| Adm | 11535 | -0.644560436 | 6.46729901 | 0.040163925 |
| Calb1 | 12307 | -0.595144561 | 9.758551905 | 8.84199E-07 |
| Polr2k | 17749 | -0.618899581 | 9.688573236 | 4.91964E-05 |
| Lcp2 | 16822 | -0.705416132 | 6.984770065 | 0.030741929 |
| Them6 | 223626 | -0.579561411 | 6.861574388 | 0.034922544 |
| Nts | 67405 | -0.703451526 | 6.234547037 | 0.046776597 |
| Gng11 | 66066 | -0.513316941 | 7.963386267 | 0.007947087 |
| Slc35f2 | 72022 | -0.544121926 | 9.088906044 | 0.001503001 |
| Gamt | 14431 | -0.568213377 | 7.247343578 | 0.024546009 |
| S1pr5 | 94226 | -0.507695152 | 7.664069594 | 0.034067295 |
| Psmg4 | 69666 | -0.505975352 | 9.434018466 | 0.006608415 |
| Ptgdr | 19214 | -1.32974355 | 4.983899453 | 0.047118142 |
| Scg5 | 20394 | -0.581842099 | 12.43550877 | 0.023621637 |
| Ooep | 67968 | -1.002554292 | 6.179510283 | 0.044605899 |
| Rgs10 | 67865 | -0.568781528 | 8.49395509 | 0.005473496 |
| Slc35f4 | 75288 | -0.568156415 | 8.396948648 | 0.01115218 |
| D830030K20Rik | 320333 | -0.514962333 | 7.245775868 | 0.042323403 |
| Isl2 | 104360 | -0.773737785 | 9.079083095 | 0.002939652 |
| Tst | 22117 | -0.549635389 | 7.404249884 | 0.032489555 |
| Tac1 | 21333 | -0.520262652 | 9.868847948 | 0.003680335 |
| Tmsb15l | 399591 | -1.215576017 | 7.267599012 | 0.025905953 |
| Tmsb15b1 | 666244 | -1.439657655 | 5.496540891 | 0.031677712 |
| Rxrg | 20183 | -0.75521305 | 7.637970014 | 0.046168942 |
| Ptgs2 | 19225 | -4.087572999 | 3.578859459 | 0.000316799 |
| Galr2 | 14428 | -3.316355822 | 2.803286718 | 0.012751635 |
| Plet1 | 76509 | -5.89668917 | 2.503135234 | 0.000314417 |
| Pstpip1 | 19200 | -5.501266439 | 2.106282333 | 0.014682691 |
| Slc6a13 | 14412 | -5.473657171 | 2.080419929 | 0.002847159 |
| 1520401A03Rik | 320309 | -5.394930575 | 2.000138082 | 0.019058167 |
| Il15ra | 16169 | -3.357543538 | 1.896007438 | 0.046500043 |
| 1700024G13Rik | 67085 | -5.282765504 | 1.88846044 | 0.003773373 |
| Glra4 | 14657 | -5.100104237 | 1.707416476 | 0.008781083 |
| Tescl | 69301 | -5.001434452 | 1.606619081 | 0.011148121 |
| Cryab | 12955 | -4.917618536 | 1.522196105 | 0.012474095 |
| Krt87 | 406219 | -4.847318021 | 1.453039013 | 0.015941958 |

- Supplementary File 3b. List of DEGs downregulated in *Isl2* null lumbar spinal cords.

| Gene symbol | Gene ID | log2.fc | log2.baseMean | raw.pval |
| --- | --- | --- | --- | --- |
| Uts2b | 224065 | -1.165851 | 8.655530693 | 1.27513E-12 |
| A730046J19Rik | 319764 | -0.7466575 | 9.387358694 | 6.79306E-09 |
| Anxa2 | 12306 | -0.5194798 | 11.5542895 | 8.05736E-09 |
| Chodl | 246048 | -0.6945365 | 10.44187696 | 1.91026E-08 |
| Kcnab1 | 16497 | -0.744645 | 10.31096017 | 1.31086E-07 |
| Slc35f2 | 72022 | -0.8264821 | 8.633217476 | 4.97829E-07 |
| Fam135b | 70363 | -0.5415007 | 11.05557423 | 1.62823E-06 |
| Etv4 | 18612 | -1.043516 | 9.247317112 | 2.84463E-06 |
| Sema3e | 20349 | -0.8609576 | 8.354985756 | 3.83509E-06 |
| Prph | 19132 | -0.8343678 | 11.48173009 | 8.87232E-06 |
| Hrk | 12123 | -0.7565686 | 9.678582293 | 6.63857E-05 |
| Dkk1 | 13380 | -2.0527489 | 5.398956184 | 8.28723E-05 |
| Barx2 | 12023 | -1.0084164 | 8.289726721 | 0.000122524 |
| C1ql3 | 227580 | -0.5344772 | 10.43901861 | 0.000125424 |
| Fam184b | 58227 | -0.6425083 | 8.308029167 | 0.000131697 |
| Ntf3 | 18205 | -0.6515647 | 9.758396412 | 0.000148653 |
| Lix1 | 66643 | -0.5468416 | 9.271161115 | 0.000216667 |
| Megf11 | 214058 | -0.5849282 | 10.58445463 | 0.000235624 |
| A830018L16Rik | 320492 | -0.5865687 | 9.401359748 | 0.000300134 |
| Moap1 | 64113 | -0.5929349 | 10.21058168 | 0.000777392 |
| Gpr149 | 229357 | -0.7348013 | 7.972135851 | 0.001004322 |
| Nos1 | 18125 | -0.8149719 | 7.28296608 | 0.001295944 |
| Isl2 | 104360 | -0.6874868 | 9.287543536 | 0.001930343 |
| Hoxc11 | 109663 | -0.7895326 | 7.880058618 | 0.00294485 |
| Tmem100 | 67888 | -0.7048196 | 7.332158136 | 0.003098591 |
| Fgd5 | 232237 | -0.5383129 | 10.13456524 | 0.006146116 |
| Far2 | 330450 | -0.5622073 | 8.427510837 | 0.007298354 |
| Ifitm2 | 80876 | -0.5286083 | 8.818494876 | 0.007368153 |
| Lrp1b | 94217 | -0.5045616 | 10.68576005 | 0.007523832 |
| Grin2a | 14811 | -0.5545144 | 9.338343892 | 0.007806205 |
| Ttn | 22138 | -0.8476447 | 8.321208369 | 0.008845867 |
| Mchr1 | 207911 | -1.3253808 | 5.59658385 | 0.00888268 |
| Adamts15 | 235130 | -0.5518801 | 7.63033408 | 0.009718125 |
| Bcl2l15 | 229672 | -1.0369575 | 5.643060254 | 0.01224537 |
| E130311K13Rik | 329659 | -0.6203851 | 7.216058093 | 0.013125999 |
| Bean1 | 65115 | -0.5342686 | 8.187712187 | 0.01489529 |
| Galr1 | 14427 | -0.7728919 | 6.487536444 | 0.018399841 |
| Gpr26 | 233919 | -0.5172011 | 7.593578593 | 0.019212964 |
| Khk | 16548 | -0.8341847 | 5.953176157 | 0.020252424 |
| Dusp2 | 13537 | -0.7461211 | 6.358554465 | 0.020914466 |
| Dqx1 | 93838 | -0.9382271 | 5.749548449 | 0.021129708 |
| Xlr3a | 22445 | -0.7633577 | 8.075987889 | 0.021470348 |
| Ephx1 | 13849 | -0.8522904 | 5.962962398 | 0.023145978 |
| Oprd1 | 18386 | -0.8222988 | 6.051686604 | 0.023226748 |
| Exd1 | 241624 | -0.6840596 | 6.375626086 | 0.023814326 |
| Arhgef37 | 328967 | -0.5298959 | 7.374048624 | 0.024575462 |
| Tmsb15b2 | 100034363 | -0.5442684 | 7.862198441 | 0.026571889 |
| Rnase4 | 58809 | -0.5717073 | 7.87582853 | 0.027207241 |
| Mcf2 | 109904 | -0.6645315 | 6.505514998 | 0.03003516 |
| Tox2 | 269389 | -0.6300334 | 6.960022504 | 0.03029796 |
| Pcp4 | 18546 | -0.6200091 | 7.78466466 | 0.031624947 |
| Kcnj12 | 16515 | -0.6537096 | 6.789239156 | 0.033138838 |
| Scg5 | 20394 | -0.6823047 | 12.65674129 | 0.034270765 |
| C3 | 12266 | -0.6351513 | 6.526927066 | 0.035327187 |
| Chrm5 | 213788 | -0.9068024 | 5.480828935 | 0.035555499 |
| Htr4 | 15562 | -0.8680494 | 5.646219922 | 0.036981295 |
| Arhgap25 | 232201 | -0.5610493 | 7.134237332 | 0.037289621 |
| Mctp1 | 78771 | -0.5976175 | 7.29831331 | 0.037441534 |
| Sertm1 | 329641 | -0.5144237 | 8.023343196 | 0.037660588 |
| Col1a2 | 12843 | -0.5838442 | 7.309494108 | 0.039065152 |
| Gabra5 | 110886 | -0.5306581 | 7.801195163 | 0.039971439 |
| Sardh | 192166 | -0.6166734 | 6.778459862 | 0.042492658 |
| Rnf128 | 66889 | -0.6652868 | 6.387479373 | 0.045985697 |
| Adra2c | 11553 | -0.8190642 | 6.09760893 | 0.046498847 |
| Ddx58 | 230073 | -0.6954094 | 6.114712449 | 0.048251012 |
| Gprin3 | 243385 | -0.849829 | 7.712753074 | 0.0483457 |
| 4930447C04Rik | 75801 | -0.6058926 | 6.454086253 | 0.048946977 |
| P2rx4 | 18438 | -0.5319944 | 6.963702327 | 0.048986446 |
| Fcrl6 | 677296 | -5.9987849 | 2.610747291 | 0.000286175 |
| Layn | 244864 | -1.4888081 | 5.338594326 | 0.001201077 |
| Pnmt | 18948 | -5.6583745 | 2.270390904 | 0.001303606 |
| Wdr63 | 242253 | -5.5381592 | 2.148371141 | 0.002048268 |
| Dppa5a | 434423 | -5.602991 | 2.214391716 | 0.002221888 |
| Plet1 | 76509 | -5.4764887 | 2.085940874 | 0.002871452 |
| Ccl28 | 56838 | -2.0605846 | 4.758612714 | 0.003953002 |
| Galnt15 | 78754 | -4.3771852 | 2.878552173 | 0.004455414 |
| Pvalb | 19293 | -1.3212631 | 4.832606439 | 0.010251357 |
| Cntnap3 | 238680 | -2.0355737 | 5.239609278 | 0.015199499 |
| B4galnt3 | 330406 | -1.1091366 | 5.265395033 | 0.016923219 |
| Scnn1a | 20276 | -3.0972085 | 2.743878605 | 0.01717541 |
| Cdhr3 | 68764 | -4.3496154 | 1.99265116 | 0.019559099 |
| Slc44a3 | 213603 | -5.3374342 | 1.94623252 | 0.020136292 |
| Rassf6 | 73246 | -1.6458672 | 4.011696998 | 0.022886502 |
| Tnc | 21923 | -1.4616895 | 4.675171744 | 0.028980409 |
| Btc | 12223 | -2.9559652 | 2.876757209 | 0.0295557 |
| Mrap2 | 244958 | -4.7129547 | 1.321699043 | 0.031568235 |
| 2310030G06Rik | 66952 | -1.3380041 | 4.665295803 | 0.03247613 |
| Fstl3 | 83554 | -1.4113028 | 4.463977809 | 0.035742313 |
| 9530053A07Rik | 319482 | -5.0159674 | 1.624630975 | 0.039303996 |
| Serpinb7 | 116872 | -4.5428645 | 1.153492963 | 0.04167034 |
| Slc16a11 | 216867 | -2.972095 | 2.878017175 | 0.042694156 |
| Cck | 12424 | -2.1276 | 3.159156212 | 0.044876482 |
| Colec11 | 71693 | -2.0035341 | 3.529635839 | 0.046174625 |
| Gm12500 | 791415 | -3.8944402 | 1.552805808 | 0.04643096 |
| Ifnlr1 | 242700 | -1.2018825 | 4.527166567 | 0.047879009 |
| Klb | 83379 | -3.4875894 | 2.037550675 | 0.049089026 |

- Supplementary File 3c. List of Top 30 enriched genes in sb.LMCl.2 subcluster.

| gene | avg_log2FC | pct.1 | pct.2 | p_val | p_val_adj | cluster |
| --- | --- | --- | --- | --- | --- | --- |
| Nkx6-2 | 0.781751674 | 0.535 | 0.026 | 3.35E-20 | 7.78E-16 | sb.LMCl.2 |
| Zfhx3 | 0.802423014 | 0.721 | 0.158 | 4.36E-20 | 1.01E-15 | sb.LMCl.2 |
| C1ql3 | 0.832863827 | 0.547 | 0.046 | 9.91E-18 | 2.30E-13 | sb.LMCl.2 |
| BC030500 | 0.948271638 | 0.837 | 0.362 | 7.88E-17 | 1.83E-12 | sb.LMCl.2 |
| Etv4 | 0.923105616 | 0.523 | 0.072 | 1.72E-15 | 3.98E-11 | sb.LMCl.2 |
| Rps9 | 0.301882344 | 1 | 1 | 2.94E-15 | 6.84E-11 | sb.LMCl.2 |
| Lhx1os | 0.971997723 | 0.93 | 0.434 | 5.88E-15 | 1.37E-10 | sb.LMCl.2 |
| Homer2 | 0.577069813 | 0.826 | 0.408 | 6.06E-15 | 1.41E-10 | sb.LMCl.2 |
| Rps15a | 0.319337696 | 1 | 1 | 3.21E-14 | 7.46E-10 | sb.LMCl.2 |
| Lhx1 | 0.756072808 | 0.872 | 0.362 | 1.03E-13 | 2.39E-09 | sb.LMCl.2 |
| A830018L16Rik | 0.566540745 | 0.674 | 0.23 | 1.78E-13 | 4.14E-09 | sb.LMCl.2 |
| Fgf10 | 0.296096923 | 0.419 | 0.039 | 3.69E-13 | 8.58E-09 | sb.LMCl.2 |
| Lypd1 | 0.715580154 | 0.488 | 0.092 | 5.36E-13 | 1.25E-08 | sb.LMCl.2 |
| Gpc6 | 0.521170539 | 0.802 | 0.336 | 7.42E-13 | 1.72E-08 | sb.LMCl.2 |
| Pappa | 0.44240937 | 0.523 | 0.118 | 7.95E-13 | 1.85E-08 | sb.LMCl.2 |
| Mnx1 | 0.499744147 | 0.872 | 0.421 | 8.18E-13 | 1.90E-08 | sb.LMCl.2 |
| Epha3 | 0.665873818 | 0.663 | 0.23 | 1.06E-12 | 2.47E-08 | sb.LMCl.2 |
| Nwd2 | 0.408827896 | 0.523 | 0.105 | 3.18E-12 | 7.38E-08 | sb.LMCl.2 |
| Pid1 | 0.60827671 | 0.779 | 0.355 | 4.20E-12 | 9.75E-08 | sb.LMCl.2 |
| Rps7 | 0.319805026 | 1 | 1 | 7.66E-12 | 1.78E-07 | sb.LMCl.2 |
| Kcnip1 | 0.616947799 | 0.884 | 0.592 | 9.09E-12 | 2.11E-07 | sb.LMCl.2 |
| Rpl32 | 0.267373771 | 1 | 1 | 1.32E-11 | 3.07E-07 | sb.LMCl.2 |
| Galntl6 | 0.44684827 | 0.663 | 0.237 | 1.63E-11 | 3.78E-07 | sb.LMCl.2 |
| Nxph1 | 0.513362029 | 0.826 | 0.461 | 1.70E-11 | 3.95E-07 | sb.LMCl.2 |
| Pik3r1 | 0.636404837 | 1 | 0.928 | 2.00E-11 | 4.65E-07 | sb.LMCl.2 |
| Gm43889 | 0.340133598 | 0.442 | 0.072 | 2.87E-11 | 6.67E-07 | sb.LMCl.2 |
| Sema5a | 0.459674252 | 0.756 | 0.349 | 3.07E-11 | 7.13E-07 | sb.LMCl.2 |
| Barx2 | 0.693525422 | 0.605 | 0.217 | 3.23E-11 | 7.49E-07 | sb.LMCl.2 |
| Kcnab1 | 0.503057446 | 0.849 | 0.434 | 5.31E-11 | 1.23E-06 | sb.LMCl.2 |
| Ret | 0.575664199 | 0.942 | 0.697 | 8.66E-11 | 2.01E-06 | sb.LMCl.2 |

(a) List of DEGs downregulated in *Isl2* null brachial spinal cords. (b) List of DEGs downregulated in *Isl2* null lumbar spinal cords. (c) List of Top 30 enriched genes in sb.LMCl.2 subcluster.
